# Safety of Pregnancy and Delivery With Shunted Hydrocephalus

**DOI:** 10.1001/jamanetworkopen.2024.34688

**Published:** 2024-09-18

**Authors:** Marie Discenza, Joanna E. Papadakis, Sarah Little, Joseph R. Madsen

**Affiliations:** 1 Department of Obstetrics and Gynecology, Brigham and Women’s Hospital, Harvard Medical School, Boston, Massachusetts; 2 Department of Neurosurgery, Boston Children’s Hospital, Harvard Medical School, Boston, Massachusetts

## Abstract

This cohort study examines the safety of cerebrospinal fluid shunts during pregnancy.

## Introduction

For women with shunted hydrocephalus reaching childbearing age, questions regarding the risks of cerebrospinal fluid (CSF) diversion shunts during pregnancy loom large. Current clinician resources cite decades’ old data with high shunt failure rates of 10% to 50%,^1,2,3,4,5,6^ which is likely worrisome to expectant patients and prompted this investigation into the safety of CSF shunts during pregnancy.

## Methods

This retrospective and multicenter cohort study was approved by the institutional review board at Boston Children’s and Brigham and Women’s Hospital, and patient consent was waived given its retrospective nature. Our reporting methods following the Strengthening the Reporting of Observational Studies in Epidemiology (STROBE) reporting guideline.

In a retrospective multicenter cohort study, a clinical research repository of obstetrical deliveries from 1989 to 2023 was queried. Patients with a billing code for childbirth, medical record mention of a CSF shunt (ie, ventriculoperitoneal [VP]), and hydrocephalus were identified. Eligibility was confirmed if shunt placement occurred before pregnancy, and a delivery note was available.

Data on prenatal and postnatal neurosurgical and obstetrical outcomes through 6 months post partum, including shunt-related symptoms, revisions, obstetrical complications, delivery characteristics, and infant outcomes were extracted. Safety, as defined in this study, pertained to the absence of shunt-related complications and adverse patient- or infant-related outcomes. Univariate comparisons using the Wilcoxon rank sum and Fisher exact tests assessed factors associated with shunt failure. R version 4.2.2 (R Project for Statistical Computing), and statistical significance was set at *P* < .05. Data were analyzed from June 2022 to January 2024.

## Results

This study included 85 of 518 068 (0.02%) pregnancies, in 60 patients delivering 90 infants. Congenital anomalies (18 [30%]) and intracranial tumors (17 [28.3%]) were the most common hydrocephalus etiologies (Table 1). Most patients (57 of 60 [95%]) had a VP shunt, which were placed primarily during childhood (38 of 57 [66.7%]), and 2 (46.7%) reported more than 1 shunt revision.

**Table 1.** Neurosurgical and Obstetric Characteristics of Pregnant Patients With Cerebrospinal Fluid Diversion Shunts

Patient characteristics	Patients, No. (%)^a^
Hydrocephalus history (n = 60)	
Etiology of hydrocephalus	
Obstructive, tumor-related	17 (28.3)
Pseudotumor cerebri	8 (13.3)
Posthemorrhagic	7 (11.7)
Myelomeningocele	6 (10)
Aqueductal stenosis	5 (8.3)
Postinfectious	4 (6.7)
Trauma	4 (6.7)
Chiari malformation	3 (5.0)
Other, congenital	2 (3.3)
Other or unknown	2 (3.3)
Craniosynostosis	1 (1.7)
Dandy-Walker	1 (1.7)
Age at initial shunt placement, median (IQR), y	16.0 (0.8-25.3)
Ventriculoperitoneal shunt	57 (95.0)
Other shunt configuration^a^	3 (5.0)
History of ≥1 shunt revision	28 (46.7)
Neurosurgical characteristics (n = 85)	
Shunting duration (at delivery), median (IQR), y	16.5 (6.8-27.0)
Concern for antenatal shunt malfunction (symptoms)	12 (14.1)
Headache	9 (10.6)
Visual symptoms	2 (2.4)
Fever	1 (1.2)
Abdominal pain	1 (1.2)
Nausea	1 (1.2)
Other	1 (1.2)
Antenatal intracranial imaging	2 (2.4)
Antenatal shunt taps	2 (2.4)
Postpartum concern for shunt malfunction, No. per delivery (%)	14 (16.5)
Headache	9 (10.6)
Shunt pain (abdominal, neck)	2 (2.4)
Visual symptoms	2 (2.4)
Nausea or emesis	1 (1.2)
Other	2 (2.4)
Postpartum shunt revisions	5 (5.9)
Time to malfunction, median (IQR), mo	3 (2-4)
Length of clinical follow-up from delivery, median (IQR), y	10.8 (3.9-19.4)
Obstetric characteristics (n = 85)	
Age at delivery, y	32 (29-34)
Full term (37-40 wk)	70 (82.4)
Late preterm (34-37 wk)	9 (10.6)
Moderate preterm (32-34 wk)	2 (2.4)
Very preterm (26-32 wk)	4 (4.7)
Pregnancy type	
Single	80 (94.1)
Twins	5 (5.9)
Delivery mode	
Cesarean	51 (60)
Vaginal	34 (40)
Antenatal obstetrical complications	
Pregnancy-induced hypertension	4 (4.7)
Gestational diabetes	9 (10.6)
Fetal growth restriction	1 (1.2)
Placenta previa	3 (3.5)
Other	3 (3.5)
Delivery or postpartum complications	
Postpartum hemorrhage	6 (7)
Need for transfusion	3 (3.5)
Wound complication	3 (3.5)
Birth weight, median (IQR), kg	3.1 (2.6-3.4)
APGAR 1, median (IQR)	8 (8-9)
APGAR 2, median (IQR)	9 (9-9)
Death (n = 90)	1 (1.1)

^a^ Including: ventriculouretal, ventriculoatrial, and lumboperitoneal shunts.

During pregnancy, 30 patients (35%) expressed worry about their shunt, and 29 patients (34%) were referred to neurosurgery to discuss risks of pregnancy and delivery. Symptoms concerning for malfunction occurred in 12 pregnancies (Table 1). Two cases (2.4%) underwent neuroimaging and 2 (2.4%) received shunt-taps to investigate symptoms of raised intracranial pressure. However, in all cases, shunt malfunction was ruled out.

Obstetrical complications were infrequent but included preterm birth (15 [17.6%]) and gestational diabetes (9 [10.6%]), among others (Table 1). Comparison of these outcomes to a nonshunted population was beyond the scope of this study. Delivery routes favored cesarean delivery (51 [60%]), of which 12 (23.5%) were attributed to clinician concern about the shunt. One infant died following acute respiratory complications unrelated to the mother’s shunt status (survival rate, 89 of 90 [98.9%]).

Across 85 deliveries, 14 (16.5%) experienced shunt-related symptoms within 6 months post partum, and 5 (5.9%) experienced confirmed malfunctions and revisions (Table 1). Postpartum malfunctions were more common in patients with adult shunt placement (OR, 4.2; 95% CI, 0.66-27.0; *P* = .13) (Table 2).

**Table 2.** Characteristics Associated With Postpartum Shunt Malfunction

Patient characteristics	Patients, No. (%)		*P* value^a^
	Postpartum malfunction (n = 5)	No malfunction (n = 80)	
Etiology of hydrocephalus			
Obstructive, tumor-related	2 (40.0)	26 (32.5)	.56
Pseudotumor cerebri	1 (20.0)	9 (11.3)	
Posthemorrhagic	NA	9 (11.3)	
Myelomeningocele	NA	9 (11.3)	
Aqueductal stenosis	1 (20.0)	6 (7.5)	
Postinfectious	NA	5 (6.3)	
Trauma	NA	4 (5.0)	
Chiari malformation	NA	4 (5.0)	
Other, congenital	NA	3 (3.8)	
Other or unknown	NA	3 (3.8)	
Craniosynostosis	1 (20.0)	1 (1.3)	
Dandy-Walker	NA	1 (1.3)	
Age at shunt placement, median (IQR), y	25 (0.4-26.0)	16 (1-21)	.72
Pediatric shunt placement <21 y	2 (40.0)	59 (73.7)	.13
Adult shunt placement ≥21 y	3 (60.0)	21 (26.3)	
Shunt type			
Ventriculoperitoneal shunt	5 (100.0)	75 (93.8)	>.99
Ventriculouretal shunt	NA	3 (3.8)	
Ventriculoatrial shunt	NA	1 (1.3)	
Lumboperitoneal shunt	NA	1 (1.3)	
History of ≥1 shunt revision	3 (60.0)	34 (42.5)	.65
Shunting duration, median (IQR), y	8 (8-29)	17 (7-27)	.95
Antenatal shunt symptoms	2 (40.0)	10 (12.5)	.14
Age at delivery, median (IQR), y	32 (31-33)	32 (29-34)	.82
Gestational age, median (IQR), wk	38 (37-39)	39 (37-40)	.41
Pregnancy type			
Single	5 (100)	75 (93.8)	>.99
Twins	NA	5 (6.3)	
Delivery mode			
Cesarean	2 (40.0)	49 (61.3)	.40
Vaginal	3 (60.0)	31 (38.8)	

^a^ Statistically significant set at *P* < .05.

## Discussion

Shunt complications during pregnancy are reported between 10% to 50% in dated literature.^1,2,3,4,6^ Our study provides a noteworthy update for patient counseling, as no patients experienced shunt malfunctions during pregnancy and nearly all had favorable obstetric outcomes.

Cesarean deliveries occurred more often than vaginal deliveries in our series, often attributable to clinician concerns about the shunt. Although literature on the ideal delivery modality is mixed, we found no evidence contraindicating vaginal birth, and some studies highlight an increased risk of abdominal infection in cesarean deliveries.^1,5^

Despite minimal risk of malfunction during pregnancy, we found a 5.9% postpartum malfunction rate, particularly among those with adult shunt placement. This is notably lower than the postpartum risk in other series (13.2% to 16.7%).^1,5^ Nonetheless, clinicians should pay special attention to postpartum malfunction when evaluating patients after birth to ensure timely neurosurgical follow-up.

Although our study spanned 3.5 decades, it is limited by its retrospective design and focus on patients delivering with a shunt. This prevented the assessment of questions such as fertility rate and an analysis of incidence because the detection rate among all cases remains unknown. Further study on the risk of CSF shunting and reproductive health is warranted.

## Notes

Supplement.
Data Sharing Statement
