# Supplementary material for: Safety of Pregnancy and Delivery With Shunted Hydrocephalus
Source: JAMA Netw Open. 2024 Sep 18;7(9):e2434688. doi: 10.1001/jamanetworkopen.2024.34688 (PMC11411382; doi:10.1001/jamanetworkopen.2024.34688)
Supplement: Supplement. — Data Sharing Statement [file jamanetwopen-e2434688-s001.pdf]

Data Sharing Statement

Data

Data available: Data from this study was gathered from the Complete Patient Data Science Repository (PDSR) Curated Data Set within the Mass General Brigham hospital system. The corresponding author has complete data on patients who met eligibility criteria in this study and takes responsibility for the integrity of the data and subsequent analyses herein. To respect patient privacy, and in conjunction with local Institutional Review Board approval, data regarding the specific patients included in this study are not publicly available. However, any questions regarding sharing data that support the findings of this study can be directed to the corresponding author upon request.
